# Supplementary figures and images for: Effects on Serum Inflammatory Cytokines of Cholecalciferol Supplementation in Healthy Subjects with Vitamin D Deficiency
Source: Nutrients. 2022 Nov 14;14(22):4823. doi: 10.3390/nu14224823 (PMC9698931; doi:10.3390/nu14224823)

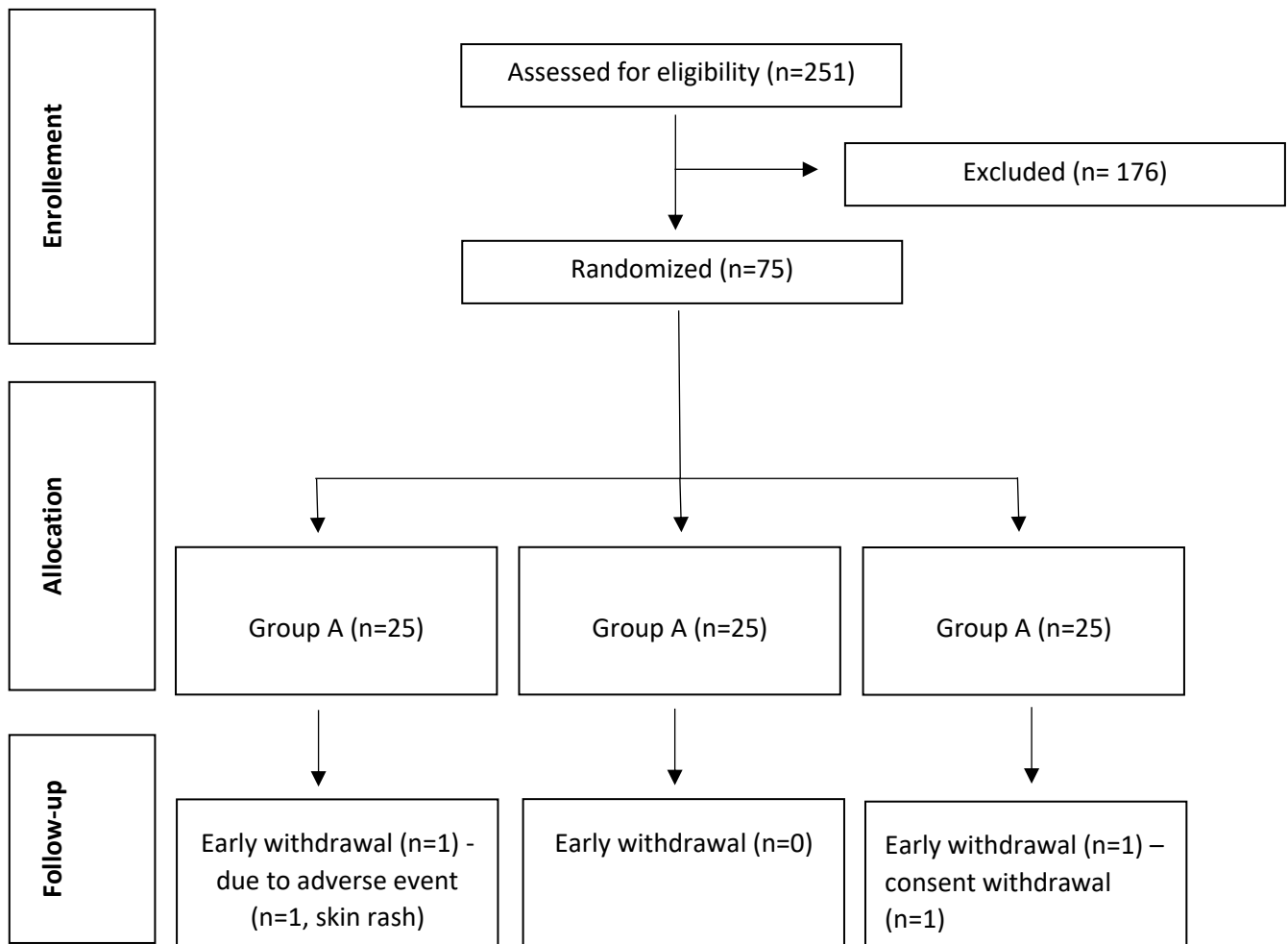

**Supplementary Figure S1.** Flowchart of the study.

Supplement: Supplementary file 1 [file nutrients-14-04823-s001.zip › nutrients-2010610-supplementary.pdf]
